# Solid water phantom heat conduction: Heating and cooling rates

**DOI:** 10.4103/0971-6203.39421

**Published:** 2008

**Authors:** Martin J. Butson, Tsang Cheung, Peter K. N. Yu

**Affiliations:** City University of Hong Kong, Department of Physics and Materials Science, Kowloon Tong, Hong Kong; 1 Illawarra Cancer Care Centre, Department of Medical Physics, Wollongong, NSW 2500, Australia; 2 Centre for Medical Radiation Physics, University of Wollongong, Gwyneville 2518, NSW, Australia

**Keywords:** Dosimetry, heat conduction, radiotherapy

## Abstract

Solid water is often the phantom material of choice for dosimetry procedures in radiotherapy high-energy X-ray and electron beam radiation calibration and quality assurance. This note investigates variation in heat conduction that can occur for a common commercially available solid water stack phantom when a temperature differential occurs between the phantom and ambient temperature. These variations in temperature can then affect radiation measurements and thus the accuracy of radiation dosimetry. In this manuscript, we aim to investigate the variations in temperature which can occur in radiation measurement incorporated (RMI) solid water phantoms, their thermal properties and the effects on radiation dosimetry which can occur because of temperature differentials. Results have shown that the rate of temperature change at a phantom center is a complex function but appears relatively proportional to the surface area of the phantom in normal clinical usage. It is also dependent on the thermal conductivity of any material in contact with the phantom; and the nature of the phantom construction, i.e., the number and thickness of slices within the phantom. A thermal time constant of approximately 20 min was measured for a 2-cm solid water phantom slice when located on a steel workbench in comparison to 60 min when located on a wooden workbench (linac couch insert). It is found that for larger solid water stack phantoms, a transient (within 1°C) thermal equilibrium exists at the center for up to 2 h, before the temperature begins to change. This is assumed to be due to the insulating properties of multiple slices within the stack, whereby very small air spaces are introduced inhibiting the heat conduction through the phantom material. It is therefore recommended that the solid water/phantom material is kept within the treatment room for closest thermal accuracy conditions or at least placed within the room approximately 10 h before dosimetry measurements. If these options are not available, a standard linear interpolation method for calculation of temperature should be used to minimize uncertainty of temperature measurements.

## Introduction

The calibration of radiotherapy radiation beams using ionization chambers requires a considerable number of correction factors to change a charge reading into absorbed dose.[1–3] One such simple factor is the temperature-pressure correction. This factor accounts for the volume of air inside an open-to-air ionization chamber and allows a correction to be applied relative to the charge collected within the chamber. The correction factor can be simply described as F(p,t) = T/T_o_ × P_o_/P, where T and P are the ambient temperature and pressure of the experimental conditions and T_o_ and P_o_ are the standard temperature and pressure respectively.[4] The value T specifically refers to the temperature inside the ionization chamber's collection volume, which is normally located in the phantom material used for calibration. Normally, a thermometer is used to record the ambient temperature for dosimetry purposes. It is normally designed to match the necessary shape, size and scale for the experimental procedure used in radiation dosimetry is performed with a solid water phantom,[5–7] a small thin thermometer is the best choice as the thermometer can be inserted into the solid water phantom's insert slot where the ionization chamber will be placed during dosimetry. The thermometer must however be removed during irradiation as the chamber fits snugly inside the solid water, and no perturbation material should be near the chamber during irradiation. A separate hole could be drilled in the phantom to accommodate a thermometer; but as many field sizes are used in dosimetry, finding a place which does not interfere with radiation beam analysis would be difficult. A thermometer can be kept outside the solid water in close proximity for temperature analysis during irradiation; however, it measures ambient room temperature. This could be considered accurate if the solid water material were to be kept in the same room and conditions as the ‘current’ ambient temperature; but in reality, two variations can commonly exist. Firstly, the ambient temperature of the treatment room may fluctuate over a short time period due to the air-conditioning system of the facility and other factors such as outside temperature or workloads. Differences up to 5°C have been observed at our institution. Internal heat sources such as a linear accelerator can also change the room temperature over a short time period by smaller amounts. Following long beam-on time, ambient room temperature near the head of a linear accelerator has been measured to increase by up to 2°C at our center. Secondly, the solid water phantom in use may not be kept in the same room or conditions as the treatment room where experimental procedures are to be performed. As such, the temperature of the solid water may differ considerably from the ambient temperature of the treatment room. Changes up to 10°C have been recorded at our center. In this case, the solid water may heat up or cool down during irradiation and be at a different temperature than that measured at the beginning of an experimental procedure or the ambient temperature of a room. These effects are quite well known but raise the question of how long it takes to reach thermal equilibrium for a solid water phantom. Solid water is constructed from epoxy resin with proprietary powder additives designed to match the electron density of water. Its physical density is 1.04 g/cm^2^, and it has a thermal conductivity of approximately 0.35 W/mK. This short note investigates the heating and cooling rates of a commonly used solid water material when moved from one temperature to another within ranges seen in a typical clinical radiotherapy department in Australia. These values are related to changes in dosimetric values obtained using standard temperature-pressure corrections over these temperature variations.

## Materials and Methods

**Figure 1:**
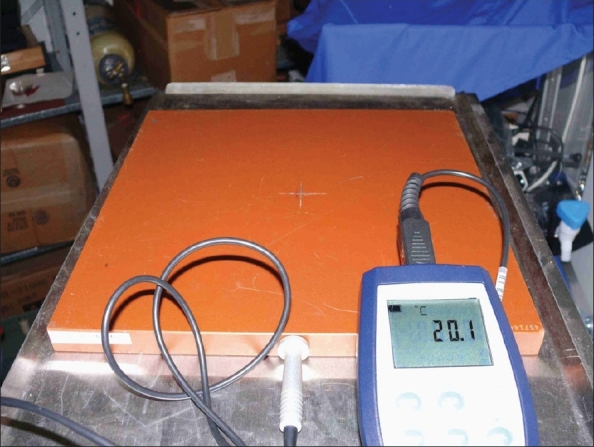
Experimental setup and measurement of temperature within the solid water phantom

An RMI solid water phantom is used for experimental verification of temperature variations due to changes in ambient temperature conditions. The solid water[8] stack consisted of a large number of 30 cm × 30 cm square solid water pieces of various thicknesses ranging from 1 mm up to 4 cm. The stack size was varied during experimental procedures from 2 cm thick up to 30 cm thick. One solid water piece (2 cm thick) has a slot drilled in the shape of a thimble ionization chamber which is used as the slice whereby the ionization chamber can precisely fit into the solid water phantom without the introduction of any other air gaps or perturbations. Experimental measurements of solid water temperature were performed by inserting a thermometer probe into this slot to the furthest point inside the phantom. The hole's extremity was subsequently taped with micro pore to minimize air flow within the slot, which would cause fluctuations in temperature measurement not necessarily present when the chamber is located in the same slot. This is shown in Figure 1. A Dual Thermo 255 digital thermometer was used, which has an accuracy of 0.5°C and a reproducibility of 0.1°C. The thermometer utilizes a thermocouple for temperature measurements. The temperature probe has two detectors, dimensions - 1-mm diameter cylinder, 2 cm long, with the thermocouple located at the end of the probes, which are attached to cables for movement of the probes. One was inserted inside the phantom as described above, and one was left outside the phantom to measure the ambient temperature conditions. This allowed a temperature variation measurement to be performed by comparing the temperature differential between the two thermometer probes. To simulate clinical temperature differentials, the solid water phantom was kept in one room and moved to another just before temperature measurements were performed. The first room had a separate air-conditioning feed and could be kept at a temperature different from that of the treatment room. The solid water phantom was kept on a wooden workbench in the treatment room (warm), while it was kept on a steel workbench in the store room (cool). The solid water phantom was also placed on a similar-thickness wooden insert when it was placed on the treatment machine for calibration purposes. Space restrictions did not allow for another standard standing bench to be used for experimental procedures. The solid water was kept at a different temperature for approximately 24 h prior to experimental measurements being performed, to allow for temperature equilibrium to occur within the solid water material. This time period was tested during the experimental procedures and was found to be adequate for temperature equilibrium for all phantom measurements performed using solid water slabs of size up to 30 cm × 30 cm × 30 cm. For experimental examination, the solid water was removed from the store room and placed in the treatment room (or vice versa), and the variation between the new ambient room temperature and that of the solid water insert position measured. This was performed with various amounts of solid water material - ranging from 2-cm thick slab up to 30-cm thick slab. Measurements were also performed while periodically removing and reinserting the thermometer into the phantom to test if any thermal effects or temperature deviations were caused by the thermometer itself. No measurable differences in measured temperature were seen, and thus these effects were considered negligible.

## Results

**Figure 2:**
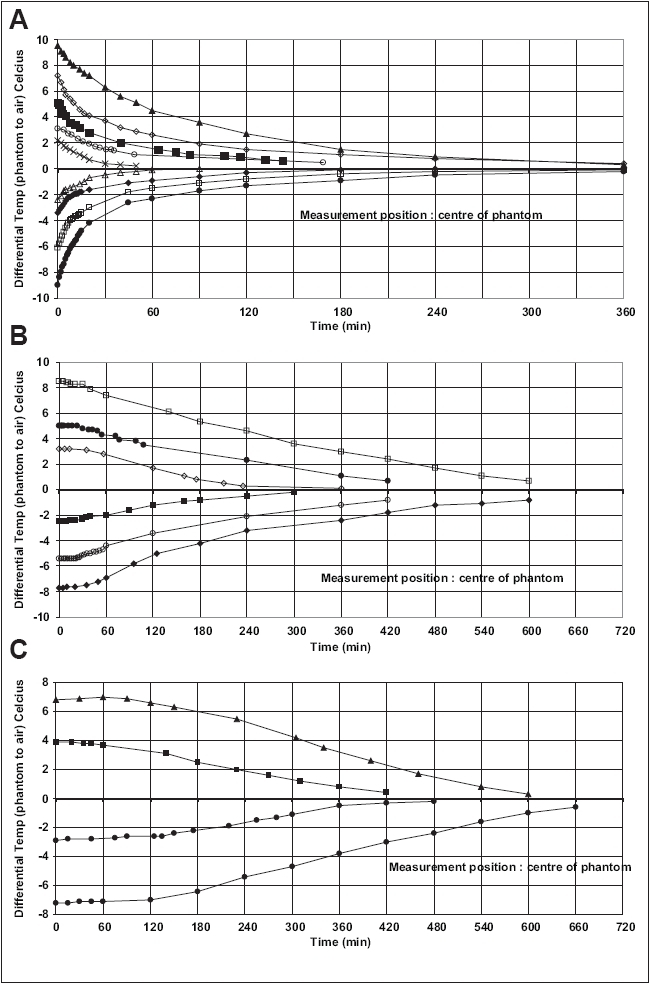
Heating and cooling rates of solid water. Shown are results for phantom sizes 2 cm × 30 cm × 30 cm (A), 10 cm × 30 cm × 30 cm (B) and 30 cm × 30 cm × 30 cm (C)

Figure 2A shows the temperature differential between the measured temperature inside the solid water phantom and the ambient room temperature for a 2 cm × 30 cm × 30 cm slab which holds a standard 0.6-cc thimble cylindrical ionization chamber. Results are shown for both positive and negative temperature differences, which relate to the solid water either being initially cooler (negative) or warmer (positive) than the ambient temperature. To note is the quick initial temperature change that occurs with the variations beginning in the first few minutes of ambient temperature change, which follows an exponential decay function. Figures 2B and 2C show similar results but for phantom sizes of 10 cm × 30 cm × 30 cm and 30 cm × 30 cm × 30 cm respectively. Figure 2B shows slower temperature variations, and the temperature at the measurement site (center of phantom) holds approximately constant for approximately 30 min before thermal changes occur. For the 30 cm × 30 cm × 30 cm phantom, the phantom center holds its initial temperature for up to 2 h before significant thermal changes occur. Up to 10 h is required for all solid water phantom arrangements to return to within 1°C of ambient temperature.

## Discussion

Heat flow through conduction can be expressed to a first-order approximation using Fourier's Law and is described by the equation

(1.1) Q=-kAdT/dx

where k = thermal conductivity, A is the cross-sectional area of the medium and T is the temperature differential. If we have two distinct temperatures at separate points within the medium, defined as T1 and T2, and the heat flow is through a material of thickness Δx, then the equation becomes

(1.2) Q=-kA(T1-T2)/Δx

This can be used to derive cooling or heating rates and is defined by Newton's law of cooling, dT/dt = −k(T1-T2) where dT/dt is the time-dependent temperature differential, k is a heat rate constant and T1 and T2 define the starting and end temperatures.

Specifically, these equations then define that the cooling or heating rates of a material in thermal contact with another body of different temperature would follow an exponential change in temperature with time and that the rate of change is proportional to the surface area of the body in question. Figure 2A shows an exponential shape for cooling and heating patterns, as expected. However, the thermal (or heating) time constant (time required for the body to change its temperature by 63.2% of its temperature span) for the heating and cooling rates is approximately 20 min (cooling) and 60 min (heating). This is assumed to be specifically due to the material the solid water is placed on during the two different procedures. When the material is cooled, it is moved from a wooden bench in the treatment bunker to a steel bench in the store room and vice versa for heating. The thermal conductivity for steel is much higher than for wood and thus would dominate the heat transfer process for cooling as shown with the thin 2-cm solid water slab. This was confirmed by measurements with the solid water slab placed on the concrete floor of both rooms, with the heating and cooling rates becoming much similar. This would complicate any efforts in modeling conduction rates; however, it highlights the effects of the thermal conductivity of the material the phantom is placed on for heating or cooling.

Figure 2B and 2C do not follow a simple exponential function for either heating or cooling in this configuration. They show an initial stationary temperature, followed by a drop. An explanation for this behavior can be found by assuming that the heat transfer process is changed within the stack phantom by the boundary surfaces of the solid water slices. The thickness of the solid water sheets within the phantom varies. Very thin air layers between the slices may act as a thermal barrier, reducing the heat transfer through the phantom to the central point where the temperature detector is placed. This was confirmed by repeating the experiment with only thick solid water slices (4-cm thick each) compared to thin sheets (size 1 mm to 5 mm thick) making up the 30 cm phantom. With a temperature difference of 8°C, the center of the thin slice phantom stayed at the same temperature for approximately 90 min compared to only 50 min for the thick slab phantom. The time required for each to reach within 1°C of ambient temperature was within 1 h for both experiments, being 11 h and 10 h respectively.

This work highlights the varying nature of heat conduction in a clinical environment for a radiotherapy phantom where the phantom material could be in contact with a number of materials of differing thermal conductivity. Ambient temperature, thermal conductivity of the phantom and material in contact with the phantom, as well as the number and thickness of slices within the phantom, all affect heat conduction. Epoxy materials (like solid water) can vary in their thermal conductivity, depending on the additives used in construction. The engineering toolbox website[9] quotes an average value of approximately 0.35 W/mK. This is compared to 0.58 W/mK for water, 0.2 W/mK for Polymethylmethacrylate (PMMA) and 0.12 W/mK for polystyrene, the other common dosimetry phantom materials. Thus an epoxy-based phantom like solid water will change temperature at a slower rate than a water phantom. However, polystyrene and PMMA phantoms will change temperature at an even slower rate than solid water. This may be of concern for medical physicists using these types of phantoms. This needs to be taken into account for the time required to reach thermal equilibrium. Our worst case scenario for temperature change occurred with a multiple thin-slice phantom placed on a wooden bench, and it took more than 10 h to reach thermal equilibrium with ambient temperature. The time required for thermal equilibrium to be achieved is then dependent on the heat transfer to the air as well as any other material in contact with it. This may mean that to achieve thermal equilibrium quicker in a clinical treatment room, a dedicated steel or metal thermal pad be utilized to speed up the heat transfer process so that the phantom material in question would reach equilibrium temperature quicker.

What does this mean for radiation dosimetry? On an average, a temperature change of approximately 3°C changes the charge reading recorded using an open-to-air ionization chamber by 1%. While variations in the order of 9°C would be uncommon in a clinical treatment room, these values have been measured as temperature differentials between our linear accelerator treatment rooms and equipment storerooms. Measurement of absorbed dose with a cool phantom (17°C) in ambient temperature of 25°C confirmed this prediction, whereby a miscalculation of 2.8% in absorbed dose would have occurred if the ambient temperature was used for calculation instead of the phantom temperature and thus ionization chamber temperature. The storeroom is kept cool (16-18°C) to aid in achieving longer life of computer equipment and longer shelf life of various accelerator parts and biomedical equipment; while the treatment room is kept warm (24-26°C) for patient comfort, with the patient being partially undressed for treatment procedures. The measurement of phantom temperatures is complicated by a complex nonlinear change associated with heating and cooling. A simple interpolation can result in a residual error due to the nonlinear temperature drift, but any interpolation is better than simply taking the initial or final temperature. In the worst case scenario measured by our experiments, the linear interpolation method performed over a 1-hour period could result in an approximate 2°C error in calculated temperature in solid water with an initial 10°C temperature differential using a 2 cm × 30 cm × 30 cm phantom size. For a phantom size of 30 cm × 30 cm × 30 cm, a maximum calculated error of approximately 1°C over a 2-hour period was found when the chamber was located anywhere within the phantom. This may be considered a reasonable uncertainty if the phantom cannot be kept inside the treatment room; however, a higher frequency of temperature measurement would be recommended. In essence, if the phantom material is kept in an area other than the measurement site, which may introduce thermal changes, the temperature measurements should be performed at short intervals within the phantom to minimize the effects of these variations. The time period of these measurements would be directly related to the amount of variation in temperature. Finally, if possible, it would be recommended to keep phantom material in the room that is used for measurements to minimize the effects of thermal variations on measurements of radiation dose calculated using open-to-air ionization chambers.

## Conclusions

It is well known that temperature variations occur between different rooms within the building in which the clinic is located, which may affect the temperature of a solid water phantom used for radiation dosimetry. This work investigated heat conduction, the heating and cooling rates of a solid water phantom, to quantify the time required for thermal equilibrium to occur within the phantom when temperature variations occur. Results show periods up to 10 h are required to allow a 30 cm × 30 cm × 30 cm solid water phantom to reach within 1°C of ambient room temperature. This was found to be relatively proportional to the phantom surface area; the thermal conductivity of materials placed in contact with the phantom; and the number and thickness of the phantom slices, which affect heat transfer within the phantom body itself. As such, it would behoove you to perform temperature measurement within the phantom material at the site of the open-to-air ionization chamber to accurately measure temperature for absorbed dose calculation. If unwanted large variations occur in ambient temperature within a hospital environment, administrators should be contacted and made aware of the difficulties that can arise in radiation dosimetry procedures. In essence, to minimize effects caused by inter-room temperature variations, phantom materials could be placed somewhere in the treatment room the day before dosimetry calibrations are performed.
